# Electroacupuncture Modulates Reproductive Hormone Levels in Patients with Primary Ovarian Insufficiency: Results from a Prospective Observational Study

**DOI:** 10.1155/2013/657234

**Published:** 2013-02-28

**Authors:** Kehua Zhou, Jingxi Jiang, Jiani Wu, Zhishun Liu

**Affiliations:** ^1^Department of Acupuncture, Guang An Men Hospital, China Academy of Chinese Medical Sciences, No. 5 Bei Xian Ge Street, Xuan Wu District, Beijing 100053, China; ^2^Department of Physical Therapy, Daemen College, 4380 Main Street, Amherst, NY 14226, USA

## Abstract

To investigate the effects of electroacupuncture (EA) on serum FSH, E2, and LH levels, women with primary ovarian insufficiency (POI) were treated with EA once a day, five times a week for the first four weeks and once every other day, three times a week, for the following two months, and then were followed up for three months. Serum E2, FSH, and LH levels were measured at baseline, at the end of treatment, and during followup. A total of 11 women with POI were included in this prospective consecutive case series study. Compared with baseline, patients' serum E2 increased, FSH decreased, and LH decreased (*P* = 0.002, 0.001, and 0.002, resp.) after EA treatment, and these effects persisted during followup. With treatment, 10 patients resumed menstruation (10/11, 90.91%), whereas one patient remained amenorrhea. During followup, two patients, including the one with amenorrhea during treatment, reported absence of menstruation. Temporary pain occurred occasionally, and no other adverse events were found during treatment. The results suggest that EA could decrease serum FSH and LH levels and increase serum E2 level in women with POI with little or no side effects; however, further randomized control trials are needed.

## 1. Introduction

Primary ovarian insufficiency (POI) is a syndrome characterized by amenorrhoea, sex steroid deficiency, and elevated gonadotrophins occurring in women under the age of 40 years. Other terms describing the disease include premature ovarian failure, premature menopause, and premature ovarian dysfunction. Clinical symptoms observed are similar to those of menopause, which include hot flashes, vaginal dryness, dyspareunia, insomnia, vaginitis, and mood swings [1]; however, this condition differs from menopause in its existence of varying and unpredictable ovarian functions [2]. Rather than complete amenorrhea in menopausal women, these patients may present with intermittent and unpredictable menses. In addition, 5–10% of women with POI will be able to conceive and deliver a child after they have received the diagnosis [3]. Consequently, a more accurate and informative term for this condition, as indicated by Nelson [2], tends to be “primary ovarian insufficiency.”

Formal diagnostic criteria of POI have not been established in any professional society; however, a commonly used definition refers to the development of amenorrhoea for four months or more due to cessation of ovarian function before the age of 40 years [4]. The diagnosis is based on elevated serum follicle-stimulating hormone (FSH) levels in menopausal range (usually above 40 IU/L) detected on at least two occasions with at least one month apart [5].

POI affects 1-2% of women younger than 40 years of age and 0.1% of women younger than 30 years of age [6]. Significant impacts including psychosocial sequelae and major health implications may result from POI. Long-term sequelae of POI include significant increase in all-cause mortality and decreased life expectancy, increased cardiovascular events, early onset of osteoporosis, increased risk of dementia or decreased cognitive function, devastating psychological effects, sexual dysfunction, and infertility [7]. Given the significant impacts and long-term sequelae of POI on patients, appropriate management is crucial in relieving symptoms and improving quality of life. Besides daily intake of calcium and vitamin D and management addressing emotional wellbeing, use of hormone (estrogen and progestin) replacement therapy for young patients remains the major treatment [2]. However, not only evidence from randomized control trials is still lacking regarding specific hormone replacement therapy (HRT) regimen, but also the long-term risks of HRT in women with POI are still unclear [8]. HRT in menopausal women was found to be associated with increased risks of breast cancer, heart attacks, and strokes [9]. Although application of these results to young women with POI may be invalid, similar risks may still exist in women with POI receiving HRT.

Acupuncture, a major component of traditional Chinese medicine, has been used in eastern Asian countries for thousands of years for various symptoms that are or are similar to those of menopause. With little to no side effects, acupuncture has been found effective in relieving hot flashes of bilaterally ovariectomized women [10], women with breast cancer [11], and women undergoing perimenopause and menopause [12]. In a recent published meta-analysis, Zheng et al. [13] summarized several previously published meta-analyses and lots of clinical trials and found that acupuncture could improve pregnancy outcomes in women undergoing *in vitro* fertilization (IVF). In addition, for patients diagnosed with polycystic cystic ovarian syndrome (PCOS), which is a disorder characterized by anovulation resulting in irregular menstruation, amenorrhea, infertility, and polycystic ovaries, Lim et al. [14] found that acupuncture may be an inexpensive effective intervention.

Although the therapeutic mechanisms of acupuncture in the above-mentioned disorders are yet to be fully investigated, a plausible hypothesis may be that acupuncture can modulate hypothalamic-pituitary-ovary axis (HPOA). Using ovariectomized rats, Chen et al. [15] found that repeated electroacupuncture (EA) increases serum E2 and reduces LH; Zhao et al. [16] found that EA stimulates hypothalamic aromatization which plays a key role in estrogen production from androgen; Ma et al. [17] reported that EA decreased the elevated estrogen receptor expression in hypothalamic preoptic area. This hypothesis is also concurred by the results from two clinical trials in which acupuncture decreased LH and increased E2 levels [10, 18]. Recent fMRI studies add further credence to the hypothesis in which acupuncture was found to modulate activities of the brain cortex including pituitary and hypothalamus [19, 20]. Nonetheless, inconsistent results were also found in other clinical trials. Dong et al. [21] found that acupuncture could significantly improve menopausal vasomotor symptoms but had no effects on serum FSH, LH, and E2 levels in menopausal patients. EA was found effective in the treatment of PCOS symptoms, but without significant change in these hormone levels [22, 23].

Similar to menopause, infertility, and PCOS, POI is also characterized by the dysfunction of HPOA. However, to our best knowledge, no studies regarding acupuncture effects on POI have been published in English; one study performed in China found positive results of acupuncture on POI [24]. Nonetheless, the study had a small sample size, lacked detailed documentation on hormone measurement, acupuncture procedures, and methods used in the assessment of efficacy [24]. In the present study, we aimed to investigate the effects of acupuncture on serum FSH, E2, and LH levels in women with POI. In addition, changes related to the availability of menstruation were also reported.

## 2. Material and Methods

### 2.1. Study Design

This was a prospective consecutive case series study performed at the Acupuncture Department of Guang An Men Hospital, China Academy of Chinese Medical Sciences. The hospital ethics committee approved this treatment protocol for women with POI, and patients signed informed consent before study participation. Acupuncture procedures were implemented by a senior acupuncturist with more than 20 years' clinical experience. Data management and analysis were performed by graduates who were blinded to the treatment procedures.

### 2.2. Participants

For inclusion, the following criteria had to be fulfilled by the patients: amenorrhea for four months or longer and FSH above 40 IU/L as detected on at least two occasions with at least one month apart. Before treatment, all patients had gone through one month baseline evaluation period during which they stopped all medications influencing reproductive hormones. These medications include but are not limited to clomiphene, human chorionic gonadotropin (HCG), Letrozole, Premarin, and Provera (Medroxyprogesterone Acetate). Patients were advised and agreed not to use these medications during study.

### 2.3. Acupuncture Protocol

Hua Tuo brand needles (size 0.45 mm × 125 mm and 0.30 × 75 mm, manufactured by Suzhou Medical Appliance, Suzhou, Jiangsu Province, China) together with GB6805-2 Electro-Acu Stimulators (Medical Supply & Equipment Co., Ltd, Shanghai, China) were used. The parameters of electric stimulation were set as the following: continuous wave with electric current frequency of 20 Hz and intensity between 1 and 4 mA based on patients' tolerance. Based on the clinical experiences of the acupuncturist, published research studies [15, 23, 24], and anatomical knowledge (direct or indirect stimulation of T12-L2, S2-S4), two EA prescriptions were used on patients alternatively. Acupoint use in Prescription 1 consists of bilateral BL33, and acupoints in Prescription 2 consist of CV4, bilateral ST25, and bilateral ST29.

Acupoints were selected and localized according to WHO Standardized Acupuncture Points Location [25]. Paired alligator clips with negative and positive electrodes of the EA apparatus were attached to the needle holders at each pair of the same acupoints on each side during treatment; no electric stimulation was used at CV4. EA stimulation in each treatment lasted 20 minutes. All patients received EA treatment once a day, five times a week for the first four weeks, once every other day, and three times a week for the following two months. They were then followed up for three months.

Needles of the size of 0.45 mm × 125 mm were inserted obliquely into the bilateral third sacral foramina (BL33) with a depth of 70–80 mm. Using 0.30 mm × 75 mm size needles, needle insertion at bilateral ST25 was performed perpendicularly with a depth of 45–55 mm. 0.30 mm × 40 mm size needles were inserted obliquely at CV4 and bilateral ST29 with a depth of 25 mm. Upon acupuncture at BL33, patients should have a strong soreness sensation which radiates to the lower abdomen. For acupuncture at ST25, CV4, and ST29, needles should be inserted just deep enough to touch the parietal peritoneum where the acupuncturist may feel resistance under the needle tip and patients may have tingling and twisting sensations.

### 2.4. Outcome Measures

At baseline of initial evaluation, the dates of patients' last menstrual cycle were documented, and patients' serum E2, FSH, and LH levels were measured. Based on patients' last menstrual cycle prior to study participation, patients' E2, FSH, and LH levels were measured on the 2nd–4th day of the 1st menstrual cycle and on the 2nd–4th day of the 4th menstrual cycle after cessation of EA treatment. In addition, patients' vaginal bleeding conditions were recorded based on patients' reports. Adverse events were documented if available.

### 2.5. Statistical Analysis

Statistical analysis was performed with the SPSS software package (Version 17.0) for Windows XP. Quantitative data of serum E2, FSH, and LH levels were expressed with mean ± SEM. Paired samples *t*-test was used to measure the difference between values at baseline, at the end of treatment, and during followup. A 5% significance level (*P* < 0.05) and two-tailed tests were used for all tests. Qualitative data including menstrual bleeding conditions and adverse events during study were described.

## 3. Results

**Figure 1:**
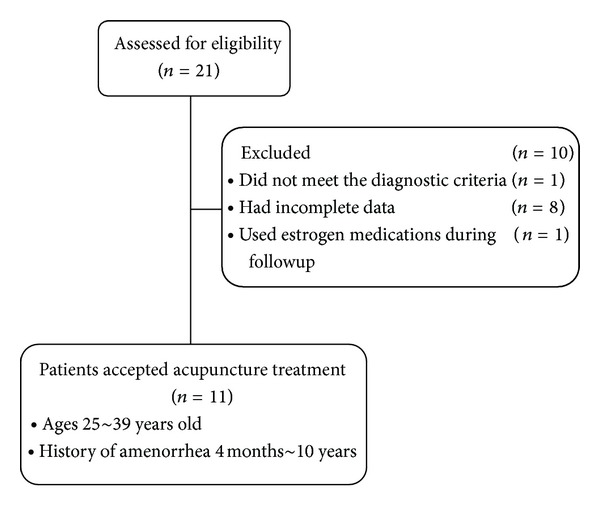
Flow chart of study participation.

From February 2, 2010 to December 30, 2011, a total of 21 patients with POI, as diagnosed by department of gynecology at tertiary-level hospitals in China, visited the Outpatient Department of Acupuncture at the Guang An Men Hospital. Of these patients, 10 were excluded from the study for the following reasons: one patient did not meet the diagnostic criteria of POI; eight had incomplete data although various patient contacts had been made; one used estrogen medications during followup (Figure 1). Of the included 11 patients, four were 25 to 30 years old, three were 31 to 35 years old, and four were 36 to 39 years old. The shortest history of amenorrhea was four months, and the longest was 10 years.

### 3.1. Hormone Levels (Tables 1 and 2)

During the one month baseline evaluation period, patients' serum E2, FSH, and LH values were 33.35 ± 10.83 pmol/L, 89.08 ± 11.97 IU/L, and 37.10 ± 3.47 IU/L, respectively. Serum E2 level increased to 223.82 ± 45.95 pmol/L after EA treatment and was maintained with a value of 217.53 ± 63.39 pmol/L during followup. Patients' serum FSH level decreased to 45.37 ± 7.07 IU/L after EA treatment and was maintained at 49.28 ± 8.85 IU/L during followup. Patients' serum LH level decreased to 22.08 ± 3.66 IU/L after EA treatment and was maintained at 22.29 ± 4.42 IU/L during followup. Compared with baseline, patients' serum E2 increased, FSH decreased, and LH decreased (*P* = 0.002, 0.001, and 0.002, resp.) after EA treatment, and these results persisted during followup (*P* = 0.016 for E2, 0.005 for FSH, and 0.023 for LH as compared with baseline). No difference was noticed in the hormone values between values at the end of treatment and during followup (*P* > 0.05).

### 3.2. Symptoms

At baseline, all patients had had no periods for more than four months and presented with varying degrees of menopausal symptoms including hot flashes, night sweats, vaginal dryness, and mood swings. With treatment, 10 patients resumed menstruation (10/11, 90.91%), whereas one patient remained amenorrhea. Of the 10 patients who regained menstruation, four patients had decreased menstrual flow but with normal color and duration as compared with normal menstrual bleeding and six regained normal duration, volume, and color of periods.

During followup, two patients reported amenorrhea including the one with amenorrhea during treatment; four patients maintained the normal menstrual cycle and flow; five patients had irregular menstrual cycles. Of the 10 patients who regained menstruation during treatment, their symptoms of night sweating, hot flashes, vaginal dryness, and mood swings, if presented, were all alleviated during treatment. During followup, their symptoms remained largely improved in six patients (6/10, 60%), but fluctuated in the other four patients (4/10, 40%). In the one patient who had had amenorrhea during the whole study, no obvious change was observed in symptoms.

### 3.3. Adverse Events

In the present study, pain which is considered normal occurred occasionally. The pain was either instant upon needle insertion or well tolerated during treatment and disappeared after needle removal. No other adverse events were reported during study.

## 4. Discussion

In the present study, EA induced significant changes of serum E2, FSH, and LH and the effects were maintained during three-month followup when acupuncture treatment was stopped. The FSH level dropped 43.71 ± 9.49 IU/L from baseline to the end of treatment (*P* = 0.001) and the decrease was still significant during the three-month followup with a value of 39.80 ± 11.19 IU/L (*P* = 0.005 as compared with baseline). E2 level increased 190.47 ± 45.10 IU/L from baseline to the end of treatment (*P* = 0.002) and the increase was still significant during the three-month followup with a value of 184.18 ± 63.47 IU/L (*P* = 0.016 as compared with baseline). These results were similar to those report by Sha et al. [24]. Using tailored cupping and moxibustion with acupuncture therapy for patients with POI, Sha et al. [24] found that FSH and E2 levels increased 30 days and 90 days after cessation of treatment whereas LH had no change 30 days but decreased significantly 90 days after cessation of treatment. The slight difference may be due to date difference of hormone measurements within the menstrual cycle as Sha et al. [24] did not reported the time of hormone measurement. Nonetheless, in the present study, 10 patients resumed menstruation, and the majority (6/10) regained normal period during treatment. The effects persisted in the majority of patients during followup. The results of the present study supported the gaining of menstruation in a top athlete recently reported in a Japanese acupuncture study [26]. In addition, improvements of other symptoms including menstrual bleeding, night sweating, hot flashes, vaginal dryness, and mood swing in the present study may also indicate certain clinical effects of acupuncture for POI.

Modulation of FSH, E2, and LH levels may help explain the mechanism of acupuncture for POI, which may be similar to the use of acupuncture in other gynecological disorders. Using acupuncture and auricular acupuncture, Zhou et al. [10] found that acupuncture could significantly increase serum E2 while decrease FSH and LH in bilaterally ovariectomized Chinese women. Although patients' exact diagnoses vary, our results match the results reported by Zhou et al. [10]. Patients included in the study by Zhou et al. [10] were 41.6 ± 5.8 years old. By referring to the diagnostic criteria of POI, we could infer that a great portion of patients included in their study may also be diagnosed with POI. Therefore, the results of the present research met the expectation as deducted from the study by Zhou et al. [10]. In addition, the results of the present study also support the effects of acupuncture in the modulation of HPOA as reported in rat studies [15–17].

Although measurement of sex hormones at a specific date of the menstrual cycle seems illogical in rats, specific measurement time of sex hormones during menstrual cycle may be accounted for the hormone differences of acupuncture effects observed in other clinical trials [21–23]. In both PCOS studies by Jadel et al. [22] and by Pastore et al. [23], hormone levels were measured during the follicular phase of the menstruation cycle when researchers did not find significant changes in E2, FSH, and LH. However, in the menopausal study, Dong et al. [21] found significant changes in these reproductive hormones with acupuncture. Our study confirmed the results reported by Dong et al. [21]. Although patients' diagnoses in these studies are different, PCOS, menopause, and POI all are characterized by dysfunctions of the HPOA and share similar physiological changes and similar clinical presentations. This is true especially for menopause and POI, and the present study of POI showed similar responses of acupuncture on reproductive hormone regulation as the study of menopause [21]. Consequently, the different results regarding reproductive hormone regulation in the PCOS studies may be due to different diagnosis or different hormone measurement time during the menstrual cycle. As patients' symptoms of PCOS were improved in both studies [22, 23], the difference may be more likely due to the later. Sex hormone was measured in the 2–4th day of menstrual phase in the present study, but measured in the follicular phase in the PCOS studies. In addition, the results of acupuncture on menstruation and gynecological symptoms in the present study partially confirmed the therapeutic effects of acupuncture on gynecological symptoms. Therefore, we should believe that acupuncture could restore the normal function of hypothalamus-pituitary-ovary axis (HPOA) in body.

Besides the HPOA hypothesis, modulation of autonomic nerve function is also proposed as the reason of acupuncture effects on gynecological disorders [27, 28]. Acupuncture stimulation to the sacral segment was found to change the vigilance state of animals via GABAergic systems suppressing the activity of noradrenergic LC neurons [27]. In the study by Wang et al. [28], researchers found that acupuncture could enhance vagal activities and suppress sympathetic activities. Based on anatomical knowledge, acupuncture at BL33 stimulates sacral nerves, part of which form the pelvic nerve and other autonomic nerve structures innervating deep organs inside the lower abdominal areas. Thus, reproductive hormone modulation effects in the present study may also be caused by the effects of acupuncture in the autonomic system.

In addition, Stener-Victorin et al. [29] found that low-frequency (2 Hz) EA stimulation with a strong intensity (6 mA) increases ovarian blood flow. The visceral peritoneum is supplied by branches from somatic efferent and afferent nerves that also supply the muscles and skin, respectively. Acupuncture deep into the parietal peritoneum at CV4, ST25, and ST29 of the present study may thus provide stimulations to the abdominal muscles and the surrounding structures and thus modulate their function and improve blood circulation inside the lower abdomen. EA stimulation parameters of 20 Hz and 1 to 4 mA based on patients' tolerance were used in the present study. As 20 Hz is significantly different from the high frequency EA (80 Hz) used in the study by Stener-Victorin et al. [29] and largest tolerant EA intensity in patients can be considered as a strong intensity, the positive results of the present study may thus partially support that middle to low-frequency EA stimulation with strong intensity may be the optimal stimulation parameters for EA treatment [29]. 

### 4.1. Limitations

Nonetheless, this study only included 11 patients at only one tertiary level hospital in China; therefore, the result of this study may not well characterize the general response of women with POI undergoing acupuncture treatments. Although the significant changes of sex hormones in the present study were most likely due to acupuncture effects; it may also be partially due to the typical disease course of POI as 5–10% of women with POI will be able to conceive and deliver a child after they have received the diagnosis [3]. With an open label prospective study design and no control group, researcher could not eliminate these cofounding factors. Furthermore, signs and symptoms of POI were subjectively reported by patients and documented by researchers rather than objectively measured with standard questionnaires and statistically analyzed. These may cause bias in the data management and documentation. Although EA at ST25, CV4, and ST29 using stimulation of 20 Hz and 1 to 4 mA based on patients' tolerance were applied in the present study, the optimal EA treatment regime with appropriate stimulations remains to be established. To test the therapeutic effectiveness of acupuncture, further randomized control trials are needed.

## 5. Conclusion

The present study demonstrated that EA could decrease serum FSH and LH levels, increase serum E2 level, and help regain menstruation in women with POI with little or no side effects; however, further randomized control trials are needed.

## Figures and Tables

**Table 1:** Demographic data and hormone values of patients during study.

Number	Age (y)	History of amenorrhea	Hormone level								
			Baseline			After treatment			Followup		
			E2	FSH	LH	E2	FSH	LH	E2	FSH	LH
			pmol/L	IU/L	IU/L	pmol/L	IU/L	IU/L	pmol/L	IU/L	IU/L
1	39	4 years	7.34	54.88	41.34	418.38	23.30	19.33	18.35	59.29	4.12
2	32	4 months	51.75	47.20	32.10	319.29	9.40	2.30	418.38	5.00	2.10
3	30	3 years	52.52	148.00	43.10	29.36	89.10	26.19	22.02	89.00	18.00
4	30	10 years	18.83	73.40	35.80	90.14	70.23	26.83	337.64	5.50	10.94
5	31	7 years	0.37	72.57	25.40	253.23	46.90	15.40	352.32	29.30	16.02
6	37	1 year	22.02	138.03	49.10	232.31	63.30	37.83	649.59	37.20	18.24
7	32	5 years	17.10	63.12	37.47	374.34	27.04	15.4	44.04	63.60	38.38
8	27	15 months	14.68	43.70	15.90	18.35	48.00	13.09	14.68	57.90	23.68
9	38	4 months	128.45	86.34	47.56	407.37	24.90	45.48	282.59	36.70	43.20
10	39	2 years	13.47	147.83	54.06	264.24	42.10	15.01	205.52	67.83	26.07
11	28	1 year	40.37	104.80	26.32	55.05	54.80	26.05	47.71	90.80	44.47

E2: estradiol; FSH: follicle-stimulating hormone; LH: luteinizing hormone.

**Table 2:** Serum E2, FSH, and LH values assessed during study (mean ± SEM).

Serum hormone level	*n*	Baseline (A)	After treatment (B)	Followup (C)	Difference *x*±*s*			*t* value			*P* value		
					AB	AC	BC	AB	AC	BC	AB	AC	BC
E2 (pmol/L)	11	33.35 ± 10.83	223.82 ± 45.95	217.53 ± 63.39	190.47 ± 45.10	184.18 ± 63.47	6.29 ± 70.25	−4.223	−2.902	0.09	0.002	0.016	>0.05
FSH (IU/L)	11	89.08 ± 11.97	45.37 ± 7.07	49.28 ± 8.85	43.71 ± 9.49	39.80 ± 11.19	3.91 ± 9.47	4.606	3.557	−0.413	0.001	0.005	>0.05
LH (IU/L)	11	37.10 ± 3.47	22.08 ± 3.66	22.29 ± 4.42	15.02 ± 3.70	14.81 ± 5.52	0.21 ± 4.31	4.057	2.683	−0.049	0.002	0.023	>0.05

E2**:** estradiol; FSH: follicle-stimulating hormone; LH: luteinizing hormone.
